# An Atypical Cause of Primary Amenorrhea: A Case Report of Rare Vaginal Agenesis

**DOI:** 10.7759/cureus.34673

**Published:** 2023-02-06

**Authors:** John P Petrykowski, Ashley C Calise, Riley A Doyle, Brent J Hurd

**Affiliations:** 1 Research, Alabama College of Osteopathic Medicine, Dothan, USA; 2 Obstetrics and Gynecology, North Alabama Medical Center, Florence, USA

**Keywords:** primary amenorrhea, imperforate hymen, mullerian agenesis, mayer-rokitansky-kuster-hauser, case report, vaginal agenesis

## Abstract

Vaginal agenesis is part of a group of anomalies, referred to as Mullerian anomalies due to their embryologic origin, in which there is a congenital absence of the vagina. We present a unique case in which a 20-year-old female presented to the Emergency Department with cyclical abdominal pain and primary amenorrhea. The original assessment showed a probable imperforate hymen; however, it was later found that she likely had vaginal agenesis. Vaginal agenesis is a rare disease, but it is prevalent enough that it should be kept at the forefront of the differential diagnosis in a woman with primary amenorrhea and recurring pain. We also highlight the importance of patient education in general, but categorically to sexual health.

## Introduction

Vaginal agenesis, also known as Mayer-Rokitansky-Kuster-Hauser (MRKH) Syndrome, is a form of Müllerian agenesis and is defined as the congenital absence of the vagina [1]. MRKH is rare, affecting one in 5,000 females [2]; it can be accompanied by either cervical or uterine agenesis. Approximately 7%-10% of women maintain either a functional uterus and endometrium that is simply obstructed or possess a rudimentary horn [1]. Embryologically, vaginal agenesis is caused by the underdevelopment of the Müllerian duct, resulting in fibrous tissue replacing the upper vagina. Patients with vaginal agenesis often present with additional urologic anomalies and skeletal abnormalities [3]. 

Presentation is often at menarche with complaints of amenorrhea and cyclic abdominopelvic pain secondary to hematometra, hematocolpos, and hematosalpinx. Often, external genitalia is normal, and a small vaginal pouch is typically seen with hymenal tissue present. Blood products retained in the uterus may be palpated on a rectovaginal exam. Ultrasonography is utilized to determine the presence and structure of the uterus, cervix, and upper vagina. MRI may be useful to determine if the endometrium is functional [1]. While first-line treatment is ideally non-surgical, the surgical creation of a neovagina is a more immediate option [4]. 

Our case is unique as it emphasizes the value of keeping a broad differential, especially in the case of primary amenorrhea, as vaginal agenesis could be the underlying cause. Additionally, the presented case highlights the importance of thorough history and physical exams when dealing with amenorrhea in young females as well as the importance of routine visits with a primary care physician in young women.

## Case presentation

We present the case of a 20-year-old female, accompanied by her mother, who initially presented to the Emergency Department (ED) with lower abdominal and back pain. Due to the nature of her pain, Obstetrics/Gynecology was consulted during her stay in the ED. The patient reported having similar pains in the past; however, she explained that it had never progressed to this level of severity, and the pain had become unbearable. She described the pain as a dull sensation that radiates to her hips and lower legs. During her time in the emergency department, she reported her pain an 8/10 in severity, which was adequately controlled with ketorolac (Toradol) and morphine. She noted associated constipation and has taken multiple doses of MiraLAX, noting one small bowel movement that day. The patient also complained of nausea and multiple bouts of emesis since the onset of her symptoms, later controlled with Ondansetron (Zofran). On review of systems, she denies any recent fever or voiding difficulties. All other systems were otherwise negative. Upon further questioning, it was discovered that the patient had never had a menstrual period. She denied routine visits with a pediatrician as well as established care with a gynecologist. Her medical history was negative for cancer, cardiovascular, endocrine, genitourinary, gastrointestinal, psychiatric, or blood disorders. Per her mother, she was up to date on all her childhood immunizations. Social history is significant for occasional alcohol use and current everyday vaping. She denied any recreational drug use. Her family history was unremarkable. The patient has two younger siblings, both of whom are healthy, per the patient’s mother. While in the ED, notable physical examination findings were significant for a firm uterus and reproducible bilateral lower quadrant abdominal tenderness with guarding. She had no tenderness of the spine with a painless range of motion. The patient displayed age-appropriate secondary sexual characteristics with no remarkable deviations.

**Figure 1:**
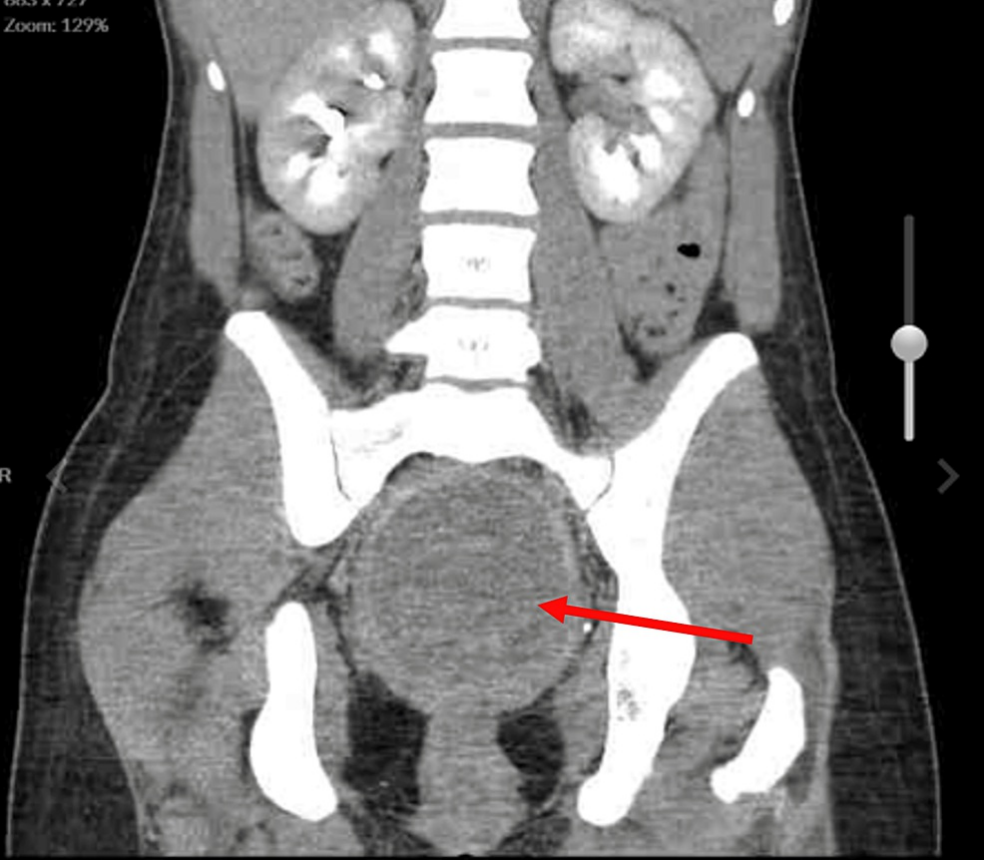
Coronal view of abdomen and pelvis.

**Figure 2:**
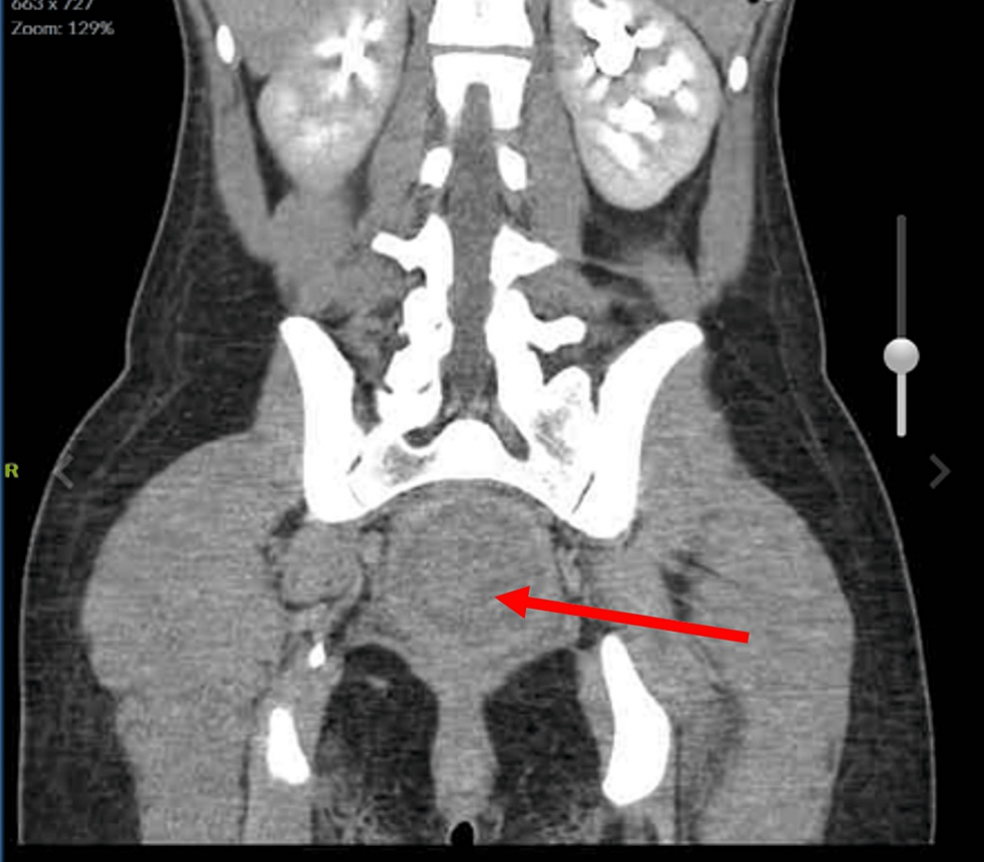
Coronal view of abdomen and pelvis.

**Figure 3:**
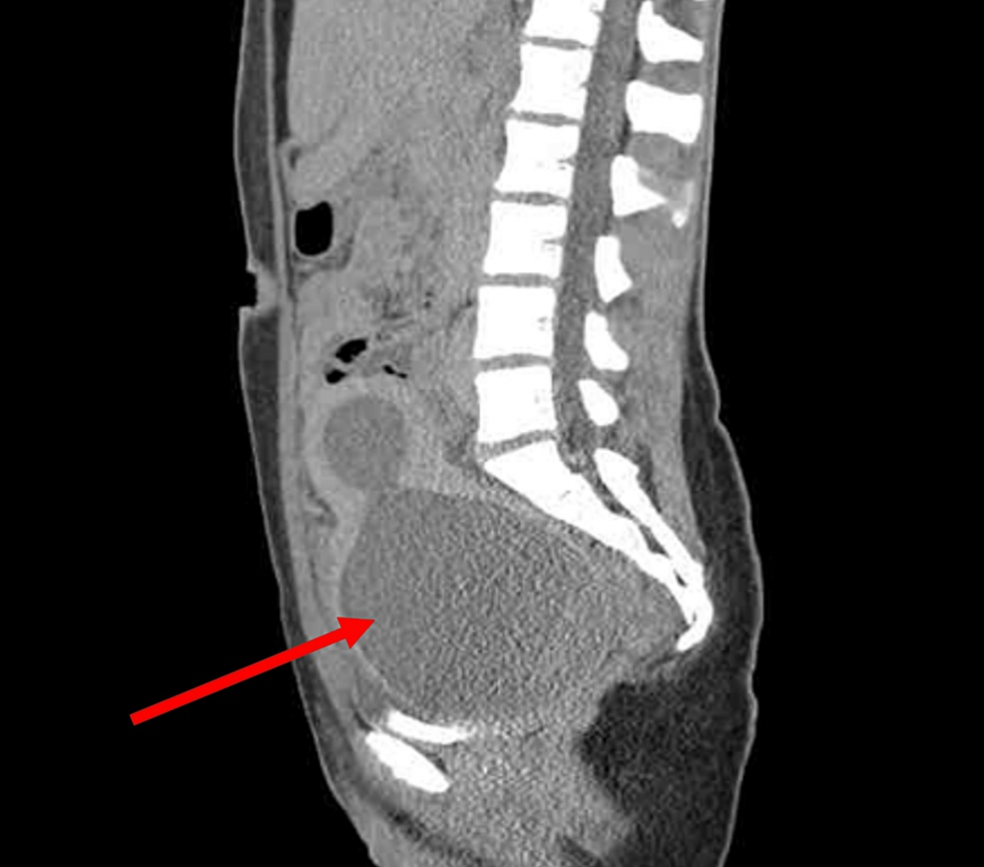
Sagittal view of abdomen and pelvis.

**Figure 4:**
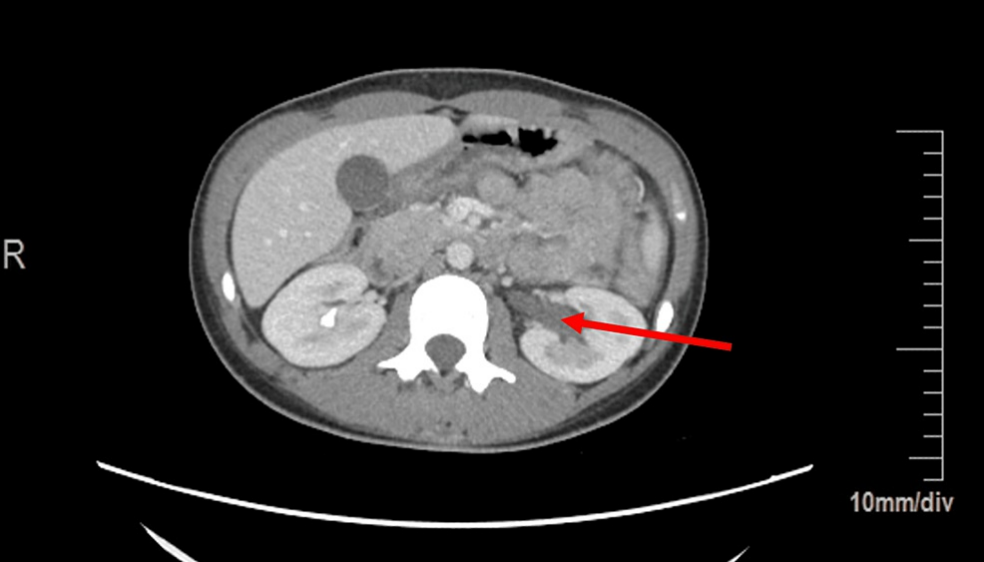
Axial view of the abdomen.

**Figure 5:**
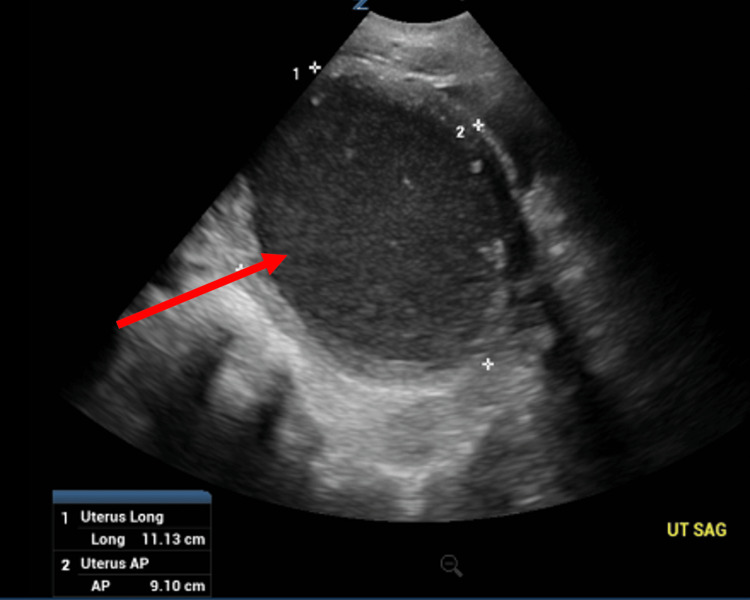
Complete pelvic ultrasound; view of the uterus.

**Figure 6:**
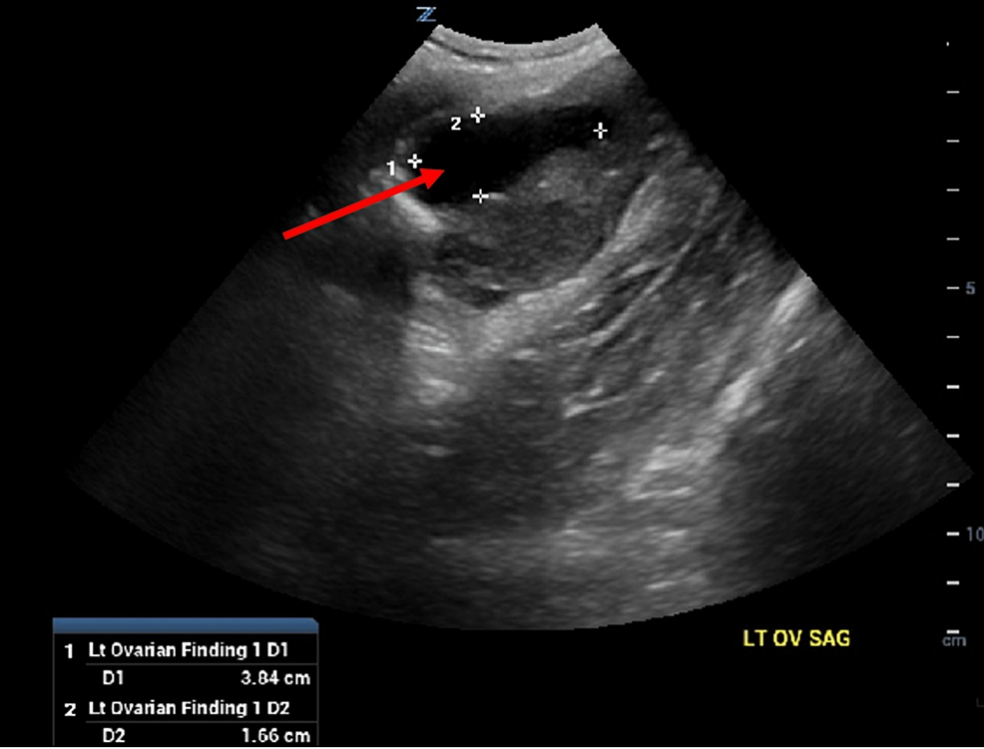
Complete pelvic ultrasound; view of the left adnexa.

The Emergency Department workup consisted of the following: urinalysis, qualitative Human chorionic gonadotrophin (hCG), Complete Blood Count (CBC) with differential, Complete Metabolic Panel (CMP), urine drug screen, lipase levels, Computed Tomography (CT) scan of the abdomen/pelvis with contrast, and a complete pelvic ultrasound. Relevant values are noted below (Table 1). The CT scan demonstrated massive dilatation of the cervix and uterus, consistent with hematometrocolpos with a possible imperforate hymen (Figures 1, 2, 3). Additionally, the CT showed mild left hydroureteronephrosis, likely secondary to the mass effect upon the distal ureter and a 3.84 cm complex cyst in the left adnexa (Figure 4). Ultrasound of the pelvis demonstrated findings consistent with hematometrocolpos as well as a physiologic left ovarian cyst (Figures 5, 6).

**Table 1:** Pertinent lab values. (H): High, (L): Low

T_max_	98.2°F	WBC	16.0 (H)	BUN	9 mg/dL
Pulse	82 bpm	Hemoglobin	12.9 g/dL	Creatinine	0.8 mg/dL
Respiratory rate	16	Hematocrit	36.4% (L)	AST	27 U/L
Blood pressure	129/78	Platelet count	397 (H)	ALT	13 U/L
Pulse oximetry	99%	Neutrophil %	78.3 (H)	Alkaline phosphatase	81 U/L
BMI	19.6 kg/m^2^	Lipase	104 U/L	Total bilirubin	1.30 mg/dL (H)

The pertinent radiographic findings were consistent with hematometrocolpos and a likely imperforate hymen and were discussed with the patient and her mother; following a discussion of the risks, benefits, and alternatives, the patient consented to surgical repair. The following morning, the patient was taken to the Operating Room (OR). Upon inspection, the hymenal ring was identified; an imperforate hymen was in place, though not in the classic bulging and purple appearance. The hymenal ring was demarcated, and a 15-blade scalpel was used to remove an oval-shaped ellipse of the center of the hymenal imperforation and sent to pathology for analysis. Once this layer was removed, there was a continuation of fibrous tissue, which would be unusual for an imperforate hymen. At this point, it was decided to terminate the procedure in fear of injuring the bladder or the rectum and uncertainty of where the septum or transverse wall was located. Any bleeding was cauterized, and then reapproximation of the hymenal ring was performed with a 3-0 Vicryl in a running manner.

Pathology results showed the vaginal hymenal center specimen to measure 2.2 x 1.9 x 1.5 cm. Grossly, it was received in formalin and had an appearance of pale tan-white vaginal mucosa and underlying tissue. The specimen fragment was sectioned, revealing a pale tan-white edematous appearing cut surface. Microscopic examination and a Hematoxylin and Eosin (H&E) Stain were performed, revealing a diagnosis of focally prominent koilocytic atypia or Vaginal intraepithelial neoplasia 1 (VAIN).

Following her surgery, given the results from pathology, it was determined that the likely diagnosis was partial vaginal agenesis. The patient was sent for further evaluation at a tertiary academic medical center. 

## Discussion

**Figure 7:**
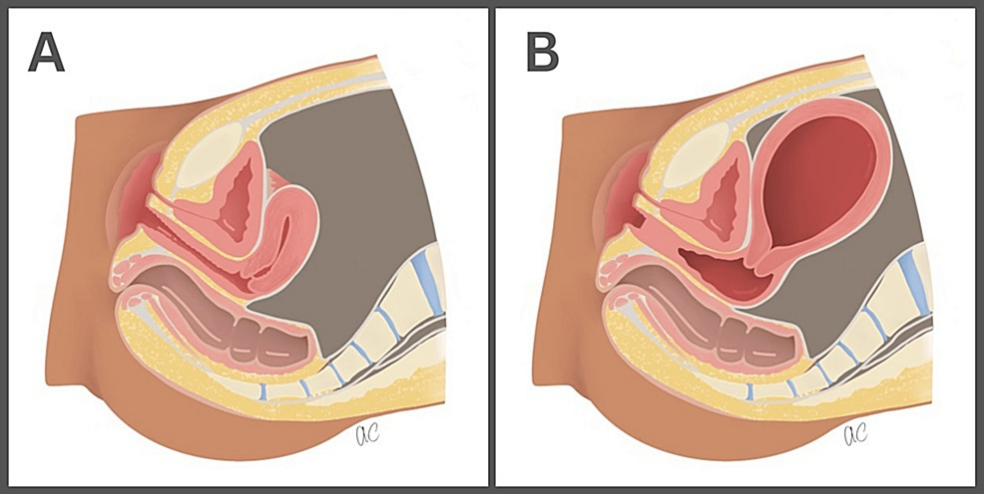
A) Normal vaginal anatomy hemipelvis. B) Vaginal agenesis of the lower vagina with retained blood products and normal external vaginal anatomy hemipelvis. Illustration by Ashley Calise.

Vaginal agenesis occurs due to the urogenital sinus failing to form the caudal portion of the vagina, which causes fibrous tissue to replace the vaginal opening during embryologic development. Vaginal agenesis falls into a group of anomalies called Mullerian abnormalities [1]. Illustrations detailing the abnormal anatomy are included below (Figure 7). While the incidence of vaginal agenesis is rare, it should always be included when a female patient presents with primary amenorrhea and cyclic abdominal pain. Most cases of vaginal agenesis are diagnosed in patients who see a gynecologist routinely; however, if patients are asymptomatic, they can present much later, as in our patient. An imperforate hymen is caused by the failure of the inferior end of the vaginal plate to form a canal. Thus, causing a membrane that extends across the hymen blocking the vaginal opening. Patients with an imperforate hymen often present with a bulge at the introitus due to the trapped menstrual blood behind the hymen. During menstruation, because of this trapped menstrual blood, patients can present with hematometra and hematosalpinx. Some presenting symptoms are cyclic abdominal pain, amenorrhea, and difficulty urinating or defecating. The incidence of an imperforate hymen is approximately one in 1,000-2,000 females and typically follows a sporadic incidence [3]. In this case, we have a 20-year-old female who presented with abdominal pain, hematometrocolpos, and primary amenorrhea, who we initially suspected had an imperforate hymen; however, post-operatively, the diagnosis of partial vaginal agenesis was suspected.

Patients with vaginal agenesis have a normal female karyotype and functional ovaries, leading to the development of secondary sexual characteristics. However, they typically present with primary amenorrhea between the ages of 15 and 17 [1]. While still rare, vaginal agenesis is second only to gonadal dysgenesis in terms of causes of primary amenorrhea, meaning it must be high on our differential diagnosis [1]. 

Diagnosis of vaginal agenesis can be done via physical exam, MRI, laparoscopy, hysteroscopy, sonography, and hysterosalpingography. A combination of these is typically used to observe the anatomy and make the diagnosis of vaginal agenesis. In a similar case, a patient presented with cyclical abdominal pain of two months onset, and on initial evaluation, she declined a genital examination. MRI of the pelvis revealed a retroflexed uterus with slight vascular engorgement, multiple small follicles in the left and right ovaries, and a 1.8 cm hemorrhagic cyst in the left ovary [5]. The patient was lost to follow-up and presented 16 months later with severe abdominal pain radiating to her back. A limited genital exam revealed only a vaginal dimple with the inability to insert a Q-tip [5]. Had this patient had the proper genital examination 16 months earlier, the diagnosis of vaginal agenesis could have been made sooner, and the appropriate treatment started at that time. Patients with vaginal agenesis often have coexisting endometriosis, which can be diagnosed using hysteroscopy or laparoscopy.

Treatment of vaginal agenesis should utilize a multi-faceted approach. It is essential to determine the underlying cause and evaluate for related congenital anomalies. Treatment of patients with vaginal agenesis involves resolving the fibrous tissue that is obstructing the vaginal opening. The American College of Obstetricians and Gynecologists (ACOG) suggests that first-line treatment is non-surgical with self-dilation due to excellent success rates [6]. Success rates with self-dilation are approximately 90% [1]. There are surgical options if the non-invasive methods fail, but these are not the first line. However, physicians should ensure that their patient is mentally and emotionally ready to endure such drastic surgical procedures.

Vaginal agenesis is a rare disease presentation, but it still occurs, and we should keep it on our differential diagnosis in females who have abdominal pain and primary amenorrhea. Many cases of vaginal agenesis are misdiagnosed or undiagnosed because of its rarity. Another major problem is the overall lack of sexual health education, creating a need for increased education pertaining to sexual health and anatomy so that anomalies such as vaginal agenesis are recognized and diagnosed earlier. Finally, this case emphasizes the importance of routine visits with a primary care physician. According to the Centers for Disease control’s Behavioral Risk Factor Surveillance System (BRFSS), nearly 10% of women report having no contact with a primary care physician [7].

As healthcare providers, we must be aware of vaginal agenesis and how to diagnose it. Vaginal agenesis can present in several different ways, and because of this, it is often misdiagnosed or undiagnosed [3]. We believe that physicians should consider vaginal agenesis in their differential diagnosis for patients presenting with cyclic abdominal pain and amenorrhea. We believe that patient education is of utmost importance, especially in the context of this condition. Here, we present a 20-year-old patient who has not experienced a menstrual cycle. As medical providers, we know that this is not normal, but without proper patient education, we cannot assume that patients know this. We believe that primary care physicians and gynecologists must provide adequate education for patients, especially when related to sexual health.

## Conclusions

Lack of routine medical care in a young woman, as in this case, can contribute to delayed diagnosis of vaginal anomalies such as vaginal agenesis. Here, we report a case of primary amenorrhea due to vaginal agenesis of the lower vagina with additional incidental findings of VAIN in a 20-year-old female, requiring surgical management. Routine care under a medical provider would have likely identified this disease presentation earlier. Thus, conveying the importance of patient education and follow-up. 
